# Immunotherapy in Ovarian Cancer: Thinking Beyond PD-1/PD-L1

**DOI:** 10.3389/fonc.2021.795547

**Published:** 2021-12-13

**Authors:** Laure Chardin, Alexandra Leary

**Affiliations:** ^1^Université Paris-Saclay, Institut Gustave Roussy, Inserm U981, Biomarqueurs Prédictifs et Nouvelles Stratégies Thérapeutiques en Oncologie, Villejuif, France; ^2^Department of Medical Oncology, Université Paris-Saclay, Institut Gustave Roussy, Inserm U981, Biomarqueurs Prédictifs et Nouvelles Stratégies Thérapeutiques en Oncologie, Villejuif, France

**Keywords:** ovarian cancer, immunotherapy, PD-L1, immunosuppression, tumor microenvironment

## Abstract

Ovarian cancer (OC) is the most lethal gynecologic malignancy, affecting approximately 1 in 70 women with only 45% surviving 5 years after diagnosis. This disease typically presents at an advanced stage, and optimal debulking with platinum-based chemotherapy remains the cornerstone of management. Although most ovarian cancer patients will respond effectively to current management, 70% of them will eventually develop recurrence and novel therapeutic strategies are needed. There is a rationale for immune-oncological treatments (IO) in the managements of patients with OC. Many OC tumors demonstrate tumor infiltrating lymphocytes (TILs) and the degree of TIL infiltration is strongly and reproducibly correlated with survival. Unfortunately, results to date have been disappointing in relapsed OC. Trials have reported very modest single activity with various antibodies targeting PD-1 or PD-L1 resulting in response rate ranging from 4% to 15%. This may be due to the highly immunosuppressive TME of the disease, a low tumor mutational burden and low PD-L1 expression. There is an urgent need to improve our understanding of the immune microenvironment in OC in order to develop effective therapies. This review will discuss immune subpopulations in OC microenvironment, current immunotherapy modalities targeting these immune subsets and data from clinical trials testing IO treatments in OC and its combination with other therapeutic agents.

## Introduction

Ovarian cancer (OC) is the most frequent cause of death among gynecological malignancies, with a 36% increase in OC incidence being expected by 2040 (source Global Cancer Observatory 2020). Optimal debulking followed by platinum-based chemotherapy remains the cornerstone of management (1). For patients with bulky stage III-IV tumors where complete resection cannot be achieved, neo-adjuvant chemotherapy followed by interval debulking surgery and adjuvant chemotherapy is a suitable alternative associated with lower morbidity (2, 3). Unfortunately, despite a response rate of 80% to first line chemotherapy, most patients subsequently relapse resulting in a five year survival rate of 45% (4). To enhance long-term disease remission, new treatment approaches are under investigation.

Immunotherapy has demonstrated great potential in treating a variety of cancers, in particular immune checkpoint inhibitors (ICI) targeting cytotoxic T-lymphocyte-associated protein 4 (CTLA-4) or Programmed death-ligand 1 (PD-L1)/Programmed cell death protein 1(PD-1) (5, 6). There is a rationale for immune-oncology (IO) treatments in OC. Many OC tumors demonstrate tumor infiltrating lymphocytes (TILs) and the degree of TIL infiltration is strongly and reproducibly correlated with survival (7, 8). A meta-analysis including 21 studies and almost 3000 patients with OC confirmed that high levels of intra-epithelial CD3^+^ or CD8^+^ T-cells were most strongly associated with both improved progression-free and overall survival (PFS and OS) (9). This positive correlation suggests that using ICIs, such as anti-PD-L1/PD-1 therapies, could be effective. Contrary to expectations, early clinical trials showed that their efficacy in OC remains limited with response rate of 10-15% and no current FDA or EMA approval (**Table 1**). PD-L1 expression has emerged as one of the biomarkers that could predict sensitivity to ICIs (15). Unfortunately expression remains rare in OC. PD-1 is mainly expressed by CD4^+^ and CD8^+^ T lymphocytes whereas its ligand, PD-L1 is widely expressed in various cell types including activated lymphocytes, fibroblasts, tumor-associated macrophages, and tumor cells. A study showed that almost two thirds of ovarian tumors demonstrated a modest expression of PD-L1 which was associated with worst prognosis, mainly on immune cells rather than tumor cells. In the IMAGYN050 trial, less than 25% of patients demonstrated >5% PD-L1+ immune cells (16). In contrast, in tumors known to be immune responsive, such as non-small cell lung cancer, PD-L1 expression ranges from 24% to 60% (17). The low response rate to PD-L1 inhibition in OC could be in part explained by the low expression level of PD-L1 on tumor cells.

**Table 1:** Results from trials exploring efficacy and safety of single-agent ICIs in OC.

Study	Trial identifier	Number of patients	ORR	PFS (Months)	OS (Months)	Ref
Efficacy and Safety of Avelumab for Patients With Recurrent or Refractory Ovarian Cancer: Phase 1b Results From the JAVELIN Solid Tumor Trial	NCT01772004	125	9.6%	10.2	11.2	(10)
A Study of Atezolizumab [an Engineered Anti-Programmed Death-Ligand 1 (PDL1) Antibody] to Evaluate Safety, Tolerability and Pharmacokinetics in Participants With Locally Advanced or Metastatic Solid Tumors	NCT01375842	12	22.2%	2.9	113.	(11)
Phase IB Study of Pembrolizumab (MK-3475) in Subjects With Select Advanced Solid Tumors	NCT02054806	26	11.5%	1.9	13.8	(12)
Efficacy and Safety Study of Pembrolizumab (MK-3475) in Participants With Advanced Recurrent Ovarian Cancer (MK-3475-100/KEYNOTE-100)	NCT02674061	376	8.0%	1.9	13.8	(13)
Safety and Antitumor Activity of Anti-PD-1 Antibody, Nivolumab, in Patients With Platinum-Resistant Ovarian Cancer	UMIN00005714	20	15%	3.5	20.0	(14)

Several studies have confirmed the relevance of using mutational and tumor antigen burden as a predictive biomarker of response to immunotherapy (18–21). However, OC is known to harbor a low neoantigen load and mutational burden (22–24). There is some data to suggest that *BRCA* mutated, or homologous recombination deficient OC may harbor higher levels of ‘personal’ neoantigens presumably due to their defective DNA repair machinery (25). In addition, OC have been reported to demonstrate cancer associated antigens such as NY-ESO-1, mutated p53, Mesothelin, MUC-16, SCP-1…which could drive immunogenecity (26). In this review we will discuss the ongoing strategies which are being explored to enhance the antitumor immune response in OC beyond PD-1/PD-L1 inhibition including: (i) combining anti PD-1/PD-L1 agents with other agents, and (ii) targeting other relevant immune subsets.

## Combination Approaches

### Poly (ADP-Ribose) Polymerase (PARP) Inhibitors and ICIs

Approximately 25% of high grade serous ovarian cancer harbor a germline or somatic mutations in the tumor suppressor genes *BRCA1* or *BRCA2* (27, 28). *BRCA1/2* mutated OC are associated with a higher lymphocyte infiltration (25, 29). BRCA1/2 are involved in DNA damage response *via* the homologous recombination (HR) pathway. HR participates in genome stability by repairing complex DNA damage such as DNA double-stranded breaks. Several studies have highlighted that *BRCA1/2* mutated OC harbor a higher number of tumor specific neo-antigenes and demonstrate increased expression of the immune checkpoint modulators, PD-1 and PD-L1, which indicated that *BRCA1/2* mutated OC may be more sensitive to PD-1/PD-L1 inhibitors (25). Unfortunately, the JAVELIN 100 trial assessing the efficacy of avelumab (anti-PD-L1) in patients with previously treated recurrent of refractory OC showed that BRCA status was not associated with clinical response (30).

Recently, increasing evidence has suggested the importance of the link between DNA damage and innate immunity (31). PARP inhibition in *Brca1*-deficient mouses elicits strong antitumor immunity *via* Stimulator of Interferon Genes (STING) pathway activation (32). PARPi induced STING activation occurs mainly in tumor cells. This pathway results in release of interferon related cytokines which in turn increase NK and other cell mediated cell killing *via* upregulation in NKG2D ligand for example. The MEDIOLA trial evaluated the combination of Olaparib (PARPi) and Durvalumab (anti-PD-L1) in *BRCA* mutated platinum-sensitive relapsed OC (33). The objective response rate was high at 71,9% but should be interpreted with caution as this response rate could be expected with a PARPi alone in *BRCA* altered OC. It is therefore difficult to conclude that there was synergistic or even additive benefit to this combination.

Combination of PARPi and anti-PD-L1 has also been tested in *BRCA* wild type OC. The combination of the PARPi niraparib and pembrolizumab (anti-PD-1) resulted in an encouraging 25% RR among patients with mainly platinum resistant *BRCAwt* recurrent OC (34).

### Anti-Angiogenic Agents and ICIs

Vascular endothelial growth factor (VEGF) is a key regulator of physiological and pathological angiogenesis and plays a major role in tumorigenesis (35). VEGF is highly expressed in OC microenvironment (36). It promotes tumor angiogenesis, enhances vascular permeability and favors peritoneal dissemination of OC through malignant ascites formation (37). In addition to its contribution to tumor angiogenesis, VEGF also has immunosuppressive properties. VEGF inhibits T-cell function, contributes to the induction and maintenance of regulatory T cells (Tregs), inhibits functional maturation of DC, enhances expression of inhibitory immune checkpoint on CD8^+^ cells and promotes tumor-associated macrophages (38). Combining anti-angiogenic agents with ICIs could reverse immunosuppression mediated by VEGF and thus increase the efficacy of ICIs in OC. *In vitro*, VEGF inhibition has been shown to enhance cytotoxic T-lymphocytes activation and down-regulate inhibitory molecules associated with T cell exhaustion (PD-L1, TIM-3, LAG-3 and CTLA-4 (39, 40).

A single-arm phase 2 study of combined nivolumab and bevacizumab resulted in a 40% and 16% response rate in platinum-sensitive and -resistant relapsed OC (41). Combination of the VEGF tyrosine kinase, Lenvatinib with pembrolizumab resulted in response rate of 29% in relapsed OC (42). Despite a sound biological rational and hints of activity in early phase trials of combined PD-L1/VEGF inhibition, a large phase III randomized clinical trial of 1^st^ line chemotherapy and maintenance bevacizumab alone or in combination with atezolizumab failed to demonstrate any benefit to the combination (16).

### Chemotherapy and ICIs

Classical cytotoxic drugs have been shown to alter the local immune state which could modulate treatment efficacy by stimulating or inhibiting the host’s anti-tumor immune response (43–45). Conventional cytotoxics may induce « immunogenic cell death », increase DC maturation, potentiate macrophage cytotoxicity and abrogate Tregs or myeloid-derived suppressor cell activity (46, 47). Two phase III clinical trials evaluated the combination of avelumab (anti-PD-L1) to standard chemotherapy and failed to show any improvement of avelumab addition in the frontline (30) or in the platinum resistant setting (48).

### Anti-CTLA-4 and ICIs

CTLA-4 is a receptor expressed on activated T cells that downregulate immune response. CTLA-4 is homologous to the T-cell co-stimulatory protein, CD28, and both molecules binds to CD80 and CD86, two co-stimulatory molecules expressed on antigen-presenting cells. Interactions of these ligands with CTLA-4 inhibits T-cell activation. Blockade of CTLA-4 with anti-CTLA-4 antibodies enhances priming and activation of naïve T-cells in lymph nodes and then migrate to tumors to cause tumor rejection. Emerging evidences suggested that combined PD-L1/PD-1 and CTLA-4 blockade could be relevant in OC. NRG GY003, a phase II trial evaluating nivolumab alone or in combination with ipilimumab, demonstrated a higher response rate (31.4%) with the combination compared to nivolumab alone (12.2%) (49). Various ongoing trials are evaluating the benefit of anti-PD-1/PD-L1 and CTLA-4 therapy including the IneOV (NCT03249142) trial which is evaluating the combination of neoadjuvant Durvalumab and chemotherapy +/- Tremelimubab (anti-CTLA-4) in 66 patients with inoperable OC. Preliminary results showed that the combination achieved an overall macroscopic complete resection rate of 58% and a rate of major pathological response (Chemotherapy Response Score 3) of 38%. However, addition of Tremelimumab did not increase CC0 or CRS3 rates (50).

## Immunosuppression in Ovarian Cancer

**Figure 1:**
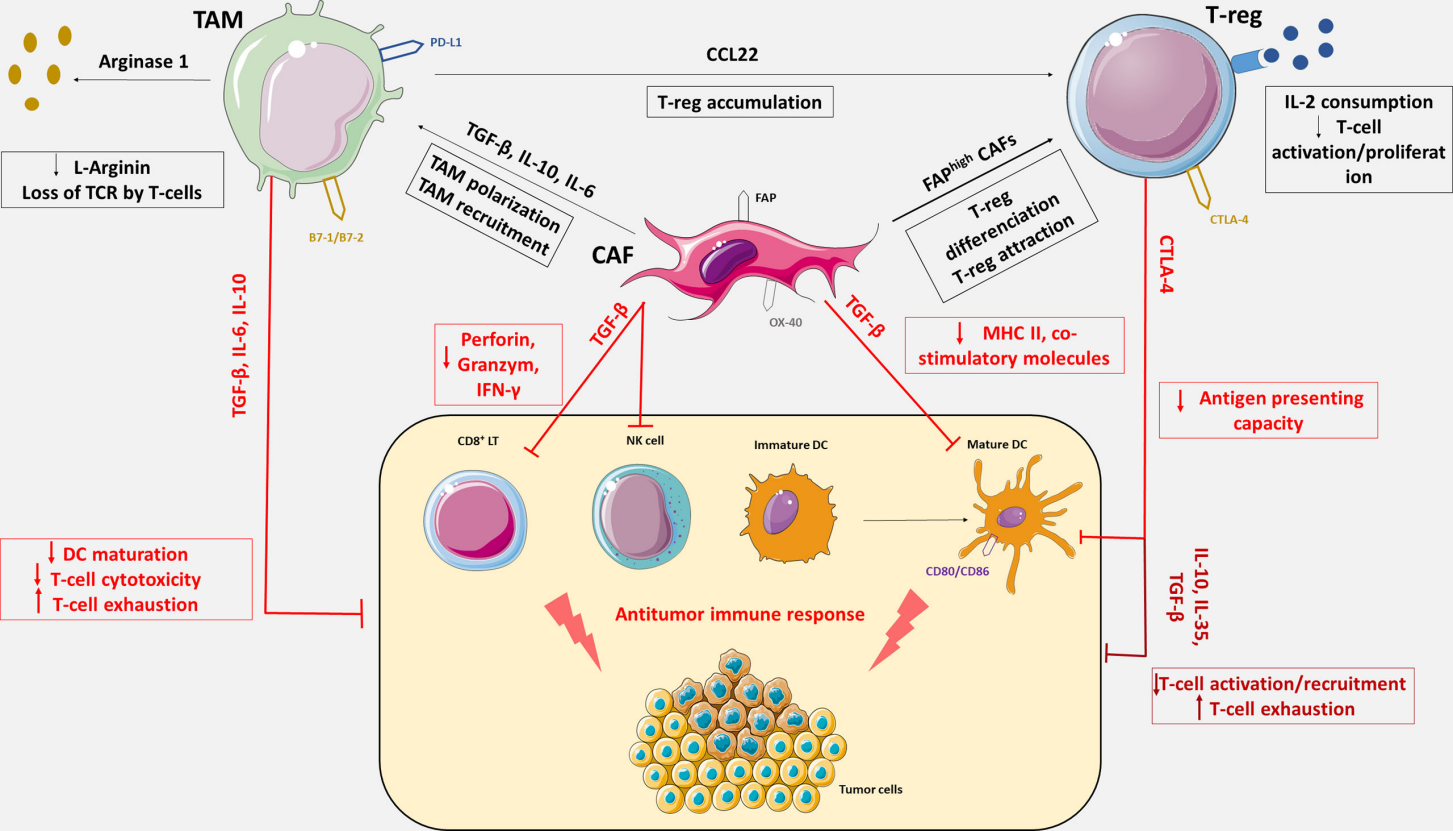
Immunosuppressive tumor microenvironment mediated by Tregs, CAFs and TAMs. OC tumor microenvironment includes antitumor immune cells such as cytotoxic CD8^+^ T lymphocytes (CD8^+^ LT), natural killer cells (NK cells) and dendritic cells (DC), and immune tolerant cells such as tumor associated macrophages, cancer associated fibroblasts and regulatory T cells responsible for immune escape. TAMs, CAFs and Tregs express an array of effector molecules that inhibit the antitumor immune responses including cell surface receptors, cytokines, chemokines, and enzymes. Through the expression of immunosuppressive cytokines including TGF-β, IL-6, IL-10 and IL-35, TAMs, CAFs and Tregs inhibit CD8^+^ LT recruitment, activation and cytotoxicity, promote CD8⁺ LT exhaustion and impede DC maturation. CAFs also reduce antigen presentation function of DC *via* the secretion of TGF-β, which downregulate the expression of MHC II and co-stimulatory molecules on DC. CAFs can secrete IL-6 and thereby contribute to monocytes recruitment and macrophages differentiation to M2-like phenotype. TGF-β expression by CAFs negatively regulate NK cells activation and cytotoxic activity. FAP^high^ CAFs increase differentiation of CD4⁺ cells into CD25^+^FoxP3^+^ Tregs and retain them at their surface by expression of OX-40. Tregs constitutively express the co-inhibitory molecule, CTLA-4 which inhibits antigen presentation by binding on CD80 and CD86, co-stimulatory molecules expresses on DC. Tregs also inhibit CD8+ LT activation *via* IL-2 consumption which is necessary to T-cells activation. The cytokine CCL22 produces by TAMs generate chemokine gradient that induces Treg accumulation in the TME. TAMs also express co-inhibitory molecules such as PD-L1 or B7-1/B7-2 and suppress CD8^+^ LT cytotoxic activity upon activation with their ligand, PD-1 and CTLA-4. TAMs also impair LT activity though metabolization of L-Arginine which is essential for T-cell function and TCR signaling.

PD-L1/PD-1 inhibition in ovarian cancer remains disappointing. CD8^+^ cells and PD-L1 may not be the only relevant immune targets in OC. Other immune subsets such as tumors-associated macrophages (TAMs), cancer-associated fibroblasts (CAFs) or regulatory T lymphocytes (Tregs) may be crucial in mediating immune tolerance and resistance (**Figure 1**) to PD-L1/PD-1 inhibition.

### Tumor-Associated Macrophages

Among the numerous factors that play a pivotal role in immunosuppressive TME of OC, TAMs are the most abundant infiltrating immune cells, particularly in malignant ascites (51–53). Due to their plastic nature, macrophages may polarize into two distinct forms depending on the local environment, the anti-tumorigenic (M1-like) and the pro-tumorigenic (M2-like).

In the OC microenvironment, TAMs generally exhibit the M2-like phenotype, with high expression of scavenger receptor class B (CD163), mannose receptor (CD206) and immunosuppressive factors, including interleukin-10 (IL-10), IL-6, TGF-β, as well as chemokines CCL18 and CCL22 to support immune escape and angiogenesis (54–56). A high density of CD163+ M2-macrophages is associated with poor prognosis in epithelial ovarian cancer whereas high ratio of M1/M2 was associated with extended survival in OC patients (57, 58).

TAMs induce an immunosuppressive environment that suppresses the function of T cells, DCs, and natural killer (NK) cells and activates the function of regulatory T cells (Tregs) (59–61). TAMs can suppress T-cell activity by the depletion of L-arginine in the tumor microenvironment. Indeed, the expression of Arginase 1 by TAMs leads to the depletion of L-arginine which is essential for T-cell functions and TCR signaling (62–64). Binding of SIRPα expressed on the surface of myeloid cells to its ligand CD47 on tumor cells acts as a « don’t eat me signal » (65). CD47 expression in patients with OC correlates with poor prognosis, potentially by inhibiting macrophage phagocytosis (66, 67).

Several strategies targeting TAMs are under investigation including: TAM depletion, TAM exclusion from the TME, TAM reprogramming from M2 to M1 and restoring phagocytic capacity.

The combination of PD0360324, a monoclonal antibody against macrophages colony-stimulating factor, with cyclophosphamide is under clinical investigation for patients with HGSOC in a phase II trial (NCT02948101).

CXCR4/CXCL12 contributes to the recruitment of the suppressive M2 macrophages and has been correlated with poor clinical outcome in OC (68–70). Pharmacological inhibition of this pathway with the CXCR4 inhibitor, AMD3100, alone or in combination with anti-PD-1 therapy have shown promising results in hepatocellular and ovarian preclinical models (71).

To restore the phagocytic capacity of TAMs, one of the most investigated strategy is the inhibition of CD47/SIRPα pathway. A number of therapeutics that target the CD47/SIRPα axis are under preclinical and clinical investigation (72). A phase I trial of an anti-CD47 antibody Hu5F9-G4 demonstrated encouraging results in OC, two patients with ovarian/fallopian tube cancers had partial remissions for 5.2 and 9.2 months (73). These results have led to an ongoing phase I trial testing Hu5F9-G4 in combination with avelumab in patients with OC (NCT03558139). BI 765063 is a SIRPα inhibitor tested in a phase I dose escalation as monotherapy or in combination with ezabenlimab (anti PD-1) in advanced solid tumors has showed promising clinical activity, including one partial response in monotherapy (hepatocellular carcinoma) and three partial response in combination (endometrial and colorectal cancer) (74).

### Regulatory T Cells

Regulatory T-cells mediate a suppressive microenvironment though the inhibition of T-cell proliferation/recruitment, cytokine production (TGF-β, IL-10, IL-35) and suppression of antigen presentation in DC in a majority of cancers (75–77). Several studies have shown that high Foxp3 expression by Tregs is associated with poor prognosis in ovarian cancer in terms of overall survival and progression-free survival (78, 79).

Denileukin Difitox (Ontak) is a recombinant fusion protein product of diphtheria toxin and IL-2 that selectively binds to CD25 on Tregs and can cause their depletion. Ontak has sown promising results in melanoma and is currently being tested in OC. In a phase I clinical trial involving seven patients with advanced adenocarcinomas, including ovarian cancers, treatment with Ontak was associated with a reduction in peripheral blood CD3+/CD4+/CD25+ cells and an increase in the number of circulating IFN-γ-producing T cells (80). On this basis, a phase II trial of Ontak in OC was initiated (81).

Toll-like receptor agonist-8 (TLR8) can reverse the suppressive function of human CD25^+^ Treg (82–84). VTX-2337, a synthetic small-molecule agonist specific to TLR8 was investigated in two phase II trials in recurrent OC (85, 86). Unfortunately, the addition of VTX-2337 to pegylated lyposomal doxorubicin did not improve clinical outcomes compared with placebo. Another potent TLR8 agonist, DN052 inhibited tumor growth and enhanced efficacy of ICIs *in vitro (*87) and is currently advancing in phase 1 trials in patients with advanced solid tumors (NCT03934359).

Tumor necrosis factor receptor 2 is expressed by highly immunosuppressive Treg and thereby represents an attractive target protein. *In vivo*, inhibition of TNFR2 leads to OC cells death, Treg inhibition and T-cells effector expansion (88). A phase I/IIa of BI-1808, a monoclonal antibody against TNFR2, as a single agent and in combination with pembrolizumab is ongoing in patients with advanced malignancies including OC (NCT04752826).

The relevance of B cell population and in particular regulatory B cells is poorly described in OC, there are some loose correlations with outcome in retrospective studies. For example CD19+ B cell populations tend to predict poor survival while CD20+ B cells predict improved PFS. However therapeutic strategies targeting B cells are currently lacking in solid tumors, especially OC (89).

### Cancer-Associated Fibroblasts

Cancer-associated fibroblasts have been implicated in tumor proliferation, invasion, metastasis, angionesis and resistance to cancer therapeutics (90, 91). More recently, some CAF subsets have been shown to dampen the anti-tumor immune response (92). CAFs secrete numerous cytokines including TGF-β, IL-6, IL-8, IL-10, and VEGF that contribute to the immunosuppressive TME by promoting monocyte recruitment or macrophages differentiation to M2-phenotype (93). Through the secretion of TGF-β, CAFs negatively regulate NK cells activation (94) and inhibit CD8+ T cell cytotoxic function by reducing the expression of perforin, granzyme and IFN-g (95, 96). CAFs also down-regulate the antigen presentation capacity of dendritic cells (97, 98) and increase differentiation of Tregs (98, 99). The capacity of CAFs to suppress anti-tumor immunity makes them another promising therapeutic target for cancer treatment. FAP (fibroblast associated protein) and α-Smooth Muscle Actine (α-SMA) are markers of a particularly immunosuppressive subpopulation of CAFs.

Numerous approaches have been investigated in pre-clinical and clinical models such as CAF depletion by targeting FAP with pharmacological inhibitors, monoclonal antibodies, DNA FAP vaccines and CAR-T cells specific for FAP (100–104).

Two types of bispecific antibody targeting FAP/IL-2(RO6874281) and FAP/4-1BB(RO7122290) are currently under investigation as CAF-targeting strategies. RO6874281 was shown to activate CD8^+^ T-cells and NK cells and to reduce Treg activity (105). The bispecific antibody targeting FAP/4-1BB enhanced T-cell stimulation *in vivo* and led to tumor remission in mouse models (106). An ongoing phase I trial is currently testing RO7122290 in monotherapy or in combination with atezolizumab in patients with advanced solid tumors (107).

Finally one last strategy is to target a ubiquitous immunosuppressive cytokine, such as TGF-β. Downregulation of TGF-β could inhibit TAMs, CAFs, Tregs as well many other immune tolerant subsets. Gemogenovatucel-T (Vigil) is an autologous tumor cell vaccine which specifically reduces expression of furin and downstream TGF-β1 and TGF-β2. Vigil was tested in a phase II trial as immunotherapy maintenance after 1^st^ line chemotherapy for advanced newly diagnosed OC (108). VIGIL showed a trend for an improved recurrence-free survival vs placebo (11,5 vs. 8,4 months); intriguingly, the benefit was significant among the subset with HR proficient tumors (RFS:10,6 vs 5,7mo, p=0,007)versus 55% for placebo, p=). Combination of Vigil with atezolizumab or durvalumab is currently being tested (NCT03073525, NCT02725489).

### Co-Regulatory Molecules

Immune cells express a variety of other co-regulatory molecules beyond PD-L1/PD-1 which could be targeted for the development of new immunotherapeutic strategies for patients with refractory tumors.

#### Co-Inhibitors

TIM-3 and LAG -3 act as negative regulators of activation and proliferation of T-cells. High expression of TIM-3 have been detected in OC and associated with poor prognosis (109, 110). In a study involving 98 patients with OC, TIM-3 was the most prevalent co-regulator with more than 75% of the samples being TIM-3 positive (111). Multiple Phase I clinical trials are currently testing anti-TIM-3 antibodies alone or in combination with anti PD-1 therapy for the treatment of cervical and ovarian cancer and advanced recurrent solid tumors (NCT03099109, NCT02608268, NCT03652077).

LAG-3 have been shown to play a important role in the development of OC (112). CD8^+^ lymphocytes co-expressing LAG-3 and PD-1 demonstrate impaired effector function and IFN production (113). Huang et al. found that LAG-3 and PD-1 inhibit T-cell signaling synergistically when they are co-expressed on TILs (114). The addition of the Anti-LAG-3 antibody, relatlimab significantly enhanced benefit from PD1 inhibition in a phase III trial in melanoma (115).

B7-H3 and B7-H4 are members of the immune regulatory ligand of the B7 family and both have been found to be overexpressed in OC (in 93% and 100% of the tumors, respectively) (116, 117). Three agents: MGD009, a dual-affinity re-targeting protein against B7-H3, DS-7300a FPA150, two antibodies targeting B7-H3 and B7-H4 respectively, are being investigated against solid tumors (NCT03406949, NCT04145622, NCT03514121).

#### Co-Stimulators

4-1BB (CD137) is a member of the TNF receptor family and mainly expressed by activated T-cells and APC. Signaling *via* 4-1BB upregulates survival genes, enhances cell division, induces cytokine production, and prevents activation-induced cell death in T cells. In OC, 4–1BB has been investigated in combination with other immune checkpoint agents such as PD-1 and TIM-3. Combination of CD137 stimulation with PD-1 inhibition in mouse ovarian cancer model induce synergistic antitumor immune response. Currently, no trial is specifically targeting 4–1BB specifically for gynecologic tumors although multiple phase I trials are under investigation in solid tumors.

OX40 (CD134), a member of the TNF superfamily, is mainly detected on active effector CD4^+^ T cell and NKT cells, as well as on Tregs. OX40 has dualistic and opposing functions depending on the cell type; it is agonist on cytotoxic T and NK cells but inhibitory on Tregs. Ramser et al. have evaluated the expression of OX40 in 47 samples of HGSOC and found that high expression of OX40 was associated with chemosensitivity and prognosis (118). Treatment with ATOR-1015, a bi-specific CTLA-4 antagonist and OX40 agonist antibody, induces T-cell activation and Treg depletion *in vitro*, reduces tumor growth and improves survival in syngeneic tumor models (119).

Glucocorticoid-induced tumor necrosis factor receptor (GITR) is predominately expressed on active B cells, NK cells, and T-cells. In a study conducted by M T Zhu et al., GITR expression in malignant cells was detected in 3.2% of OC (120). Combination of PD-1 blockade and GITR triggering showed promising results in murine ID8 OC, with 20% of the mice becoming tumor-free 90 days after tumor injection. Combined treatment with anti-PD-1/GITR antibody and chemotherapeutic drugs further increased the antitumor efficacy with 80% of mice achieving tumor-free long-term survival (121).

## Conclusion

Immune checkpoint inhibitors are some of the most prominent agents that strengthens the activity of our adaptive immune system, and have demonstrated success in treating different types of cancer. With significant promises in melanoma and other solid tumors, ICIs have also been evaluated in OC. Contrary to expectations, their efficacy for treating OC is very low.

OC’s immunosuppressive TME may contribute to the limited activity of ICIs. Moreover, CD8+ cells and PD-L1 may not be the only relevant immune targets in OC. Targeting other immune subsets such as TAMs, Tregs or CAFs may be relevant to make progress in cancer immunotherapy. In addition to the PD-L1/PD-1 axis, other immunosuppressive molecules, such as CTLA-4, TIM-3 and LAG-3 should be taken into consideration for the development of new immunotherapeutic strategies. Finally, although not the subject of this review, other promising strategies include next generation approaches such as TCR engineering, CAR-T cells, dendritic vaccination, TILs based therapies or oncolytic viruses.

## Author Contributions

Both authors contributed to the conception, writing, and editing.

## Funding

LC PhD student funded by educational grant from ARCAGY-GINECO.

## Conflict of Interest

AL: fees to institution for ad boards with AZ, Clovis, MSD, GSK, Ability, Merck Serono. AL: support to institution for clinical trials from Roche, GSK, AZ, Agenus, Iovance, Pfizer, MSD, Incyte.

The remaining author declares that the research was conducted in the absence of any commercial or financial relationships that could be construed as a potential conflict of interest.

## Publisher’s Note

All claims expressed in this article are solely those of the authors and do not necessarily represent those of their affiliated organizations, or those of the publisher, the editors and the reviewers. Any product that may be evaluated in this article, or claim that may be made by its manufacturer, is not guaranteed or endorsed by the publisher.
